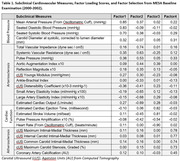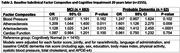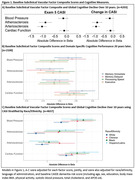# Subclinical Vascular Disorders in Relation to Cognitive Decline and Impairment: The Multi‐Ethnic Study of Atherosclerosis

**DOI:** 10.1002/alz.089209

**Published:** 2025-01-09

**Authors:** Jordan E. Tanley, Byron C Jaeger, Haiying Chen, Marc D. Rudolph, Bonnie C. Sachs, Clara Li, Katya Rascovsky, Jose A. Luchsinger, Kathleen M. Hayden, Jingzhong Ding, Yongmei Liu, Alain G. Bertoni, Susan R. Heckbert, Wendy Post, Moyses Szklo, Samuel N. Lockhart, Timothy M. Hughes

**Affiliations:** ^1^ Wake Forest University School of Medicine, Winston‐Salem, NC USA; ^2^ Department of Psychiatry, Icahn School of Medicine at Mount Sinai, New York, NY USA; ^3^ Penn FTD Center, Perelman School of Medicine, University of Pennsylvania, Philadelphia, PA USA; ^4^ Columbia University Medical Center, New York, NY USA; ^5^ Wake Forest University School of Medicine, Winston Salem, NC USA; ^6^ Wake Forest School of Medicine, Winston‐Salem, NC USA; ^7^ University of Washington, Seattle, WA USA; ^8^ Johns Hopkins Medicine, Baltimore, MD USA; ^9^ Johns Hopkins Bloomberg School of Public Health, Baltimore, MD USA

## Abstract

**Background:**

Vascular disorders are proposed as modifiable risk factors for dementia; yet, physiologic mechanisms connecting vascular disorders to cognitive impairment remain unknown. We examined subclinical cardiovascular measures to determine which predict global cognitive decline and domain specific cognitive impairment and point to potential pathways linking subclinical vascular disease and dementia.

**Methods:**

MESA includes a diverse cohort of 6,814 participants free from clinical cardiovascular disease with follow‐up over 6 clinical examinations and annual follow‐up calls. At baseline in 2000‐2002, 24 subclinical vascular measures were collected (e.g., blood pressure, carotid distensibility, artery elasticity index, heart rate, coronary calcification, carotid intima media thickness) that prior work generalized into four uncorrelated factor composites representing: atherosclerosis, arteriosclerosis, blood pressure, and cardiac function (Table 1). During Exam 5, participants began cognitive testing with the Cognitive Abilities Screening Instrument (CASI), which was repeated 10 years later (2010‐2012 to 2019‐2021) and modeled using linear mixed effects models. At Exam 6 (2019‐2021), the Uniform Data Set v3 battery was administered. Scores were normed, grouped into domains (e.g., Memory, Processing Speed, Executive Function) and modeled using generalized linear models. Cognitive impairment was adjudicated as mild cognitive impairment (MCI) or probable dementia and modeled using logistic regression. Results are reported as beta estimates or odds ratios and 95% confidence intervals (95%CI) including adjustment for baseline CAIDE dementia risk score (Figure 1), race/ethnicity, and language.

**Results:**

Baseline arteriosclerosis and atherosclerosis were independently associated with greater cognitive decline 10 years later (Figure 1a), lower domain specific cognitive performance (Figure 1b) and higher odds of cognitive impairment 20 years later (Table 2) after multi‐variable adjustment. One standard deviation increase in baseline arteriosclerosis was associated with modestly increased odds of MCI and almost two fold increased odds of probable dementia. Baseline cardiac function and blood pressure were not significantly associated with cognitive decline or impairment beyond CAIDE. Cognitive decline results were generally consistent across racial and ethnic groups (Figure 1c, p‐interaction>0.10).

**Conclusions:**

These findings suggest that arteriosclerosis and atherosclerosis are more consistently associated with cognitive impairment and global cognitive decline than cardiac function and blood pressure in a diverse population with 20 years of follow‐up.